# Challenges and management of iliac-to-femoral artery injuries in endovascular aneurysm repair

**DOI:** 10.1016/j.tcr.2026.101405

**Published:** 2026-05-25

**Authors:** Antonio Marzano, Enrico Sbarigia, Carola D'Amico, Vincenzo Brizzi, Ombretta Martinelli, Simone Cuozzo

**Affiliations:** a Unit of Vascular Surgery, Department of Cardiovascular Sciences Fondazione Policlinico Universitario A. Gemelli IRCCS, Rome, Italy; b Vascular Surgery Unit, "Sapienza" University of Rome, Department of General Surgery, Surgical Specialties and Anesthesiology, Rome, Italy; c Vascular Surgery Department, Bordeaux University Hospital (CHU), Bordeaux, France

**Keywords:** Iliac artery injuries, Branched endovascular aneurysm repair, Large-bore access complications, Vascular surgical procedures

## Abstract

**Background:**

Iatrogenic injuries to iliac-to-femoral arteries during complex endovascular procedures remain serious and potentially life-threatening. These events are influenced by adverse anatomy, procedural difficulties, and prolonged device manipulation. This report assesses the occurrence and risk factors associated with arterial disruption during endovascular thoracoabdominal aortic aneurysm (TAAA) repair and highlights adjunctive techniques that may optimize outcomes in challenging iliac anatomies.

**Case report:**

A 70-year-old woman underwent TEVAR and iB-EVAR with an *E*-nside stent-graft for a type I TAAA. Preoperative imaging confirmed anatomy suitable for an off-the-shelf device despite a narrow (6.8 mm) and tortuous (X = 1.48) external iliac artery (EIA). Because of comorbidities, a surgical conduit was reserved as a bailout if advancing the 24 F devices proved difficult. Adjunctive maneuvers, including retroperitoneal mobilization of the EIA to straighten the vessel and multiple serial dilations, were preferred. The EIA accommodated both the thoracic stent-graft and the *E*-nside system. Difficult catheterization of target vessels prolonged operative time and increased the duration of device presence within the artery. During sheath withdrawal, unexpected resistance caused hemodynamic instability due to EIA rupture, requiring emergent iliac-to-femoral bypass. Postoperative imaging showed loss of patency of the celiac trunk and left renal artery, prompting secondary occlusion of the corresponding side branches. Three-year follow-up confirmed aneurysm sac shrinkage without endoleaks.

**Conclusion:**

Well-documented risk factors increase the likelihood of EIA injury, highlighting the need to balance patient comorbidities, anatomical challenges, and therapeutic strategies. Standardized protocols are essential for managing large-bore access, and refinements in delivery system design may further enhance procedural safety and effectiveness.

## Highlights

• Iliofemoral avulsion during iB-EVAR highlights large-bore access risks.
• Narrow tortuous iliac anatomy increased risk of device-artery interaction.
• Adjunctive maneuvers enabled graft delivery without planned iliac conduit.
• Sheath withdrawal resistance led to catastrophic external iliac rupture.
• Case stresses need for standardized protocols for hostile iliac access.

## Introduction

Complex endovascular aortic aneurysm repairs using off-the-shelf stent-grafts are standard and widely practiced procedures. However, the success of these interventions relies on a comprehensive understanding of the large-bore access required for safe device delivery and withdrawal. Anatomical challenges, including vessel tortuosity, narrow arteries, and extensive calcification, can extend operative times and increase the duration of the delivery system's presence within the artery, thereby elevating the risk of access-site related complications. Although largely under-reported, the incidence of vascular access complications ranges from 4.3 to 15.9%, occurring with similar frequency in the femoral and iliac arteries, while involvement of the distal aorta remains rare.

In such cases, adjunctive maneuvers, such as surgical cutdown to common femoral artery (CFA), retroperitoneal mobilization of the external iliac artery (EIA), or continuous retrograde flushing with saline solution, may mitigate the risks of unforeseen interaction between the graft and arterial walls, optimizing outcomes. This case report underscores the need for standardized surgical protocols to systematically assess and address these anatomical challenges in procedures involving large-bore devices, aiming to prevent life-threatening complications, such as arterial disruption.

## Case report

A 70-years-old woman was admitted to our Vascular Surgery Unit due to an asymptomatic type I thoraco-abdominal aortic aneurysm (TAAA) with a maximum transversal diameter of 64.1 mm.

The patient's medical history reported a life-long smoking habit, chronic obstructive pulmonary disease (COPD), previous lung cancer, mild obesity (BMI 27.5 kg/m^2^), hypertension, dyslipidemia, and rheumatoid arthritis.

Due to multiple comorbidities, the patient was deemed unsuitable for open repair and was instead considered for an endovascular approach.

**Fig. 1:**
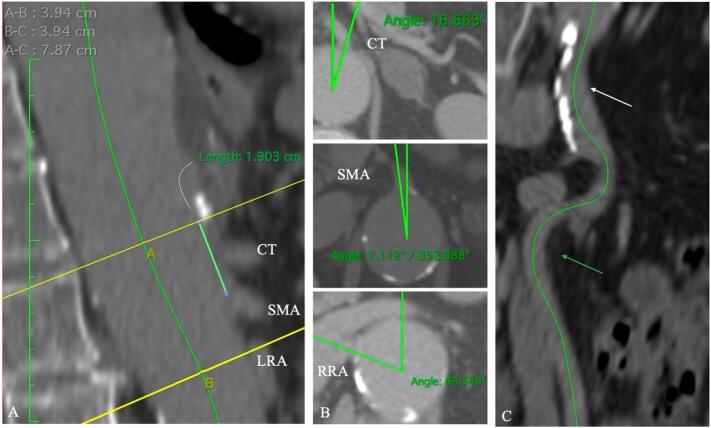
Anatomical features of the preoperative computed tomography angiography (CTA). A) The A-line passed through the ostium of the celiac trunk (CT), whereas the B-line passed through the ostium of the left renal artery (LRA). The distance between the CT and the superior mesenteric artery (SMA) was 19.3 mm, while the distance between the CT and the LRA was 39.4 mm; B) Angulation of the ostia of the visceral vessels relative to the major longitudinal axis passing through the aorta was measured as follows: CT: 16.6°, SMA: 352.8°, LRA: 82°, right renal artery (RRA): 292°; C) Right iliac axes features, with minimal calcifications at the level of the common iliac artery (white arrow) measuring 2.1 mm in thickness and an absence of calcification at the level of the external iliac artery (green arrow). (For interpretation of the references to colour in this figure legend, the reader is referred to the web version of this article.)

**Fig. 2:**
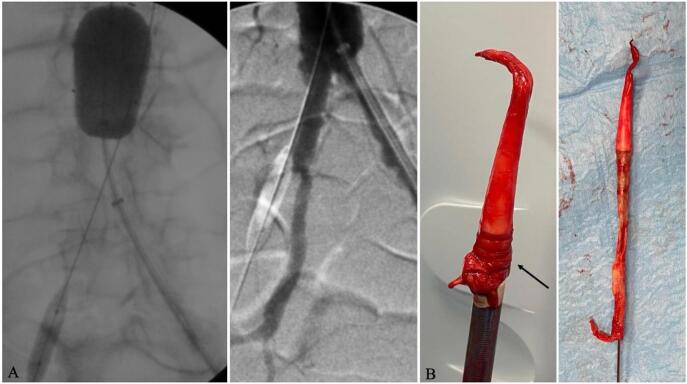
A) Coda balloon (Cook Medical Inc., Bloomington, Ind, USA) placed in the distal infrarenal aorta to control blood flow; B) Avulsed iliac-to-femoral artery attached at the 24 F sheath of the *E*-nside stent-graft. The artery is curled at the interface between the tapered tip and the sheath of the delivery system (black arrow). This curling suggests mechanical strain induced by the spatial discontinuity at the gap, potentially exacerbated by the interaction delivery system-arterial wall due the extension of operative time.

**Fig. 3:**
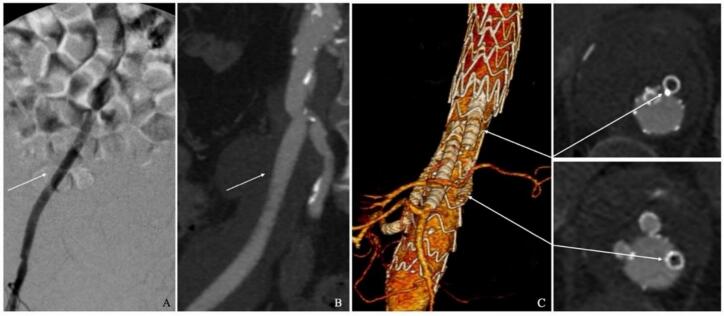
Digital Subtraction Angiography (DSA, 3 A) and post-operative Computed Tomography Angiography (CTA; 3B) revealed no extravasation of dye and excellent flow in the prosthetic iliac-to-femoral bypass (white arrows), with the patency of the internal iliac artery. C) Amplatzer Vascular Plugs I (discs' diameter of 12 mm) at the level of the inner branch of celiac trunk (CT) and a Cera Vascular Plug (discs' diameter of 10 mm) at the level of the inner branch of RRA (white arrows), without signs of endoleaks.

Preoperative computed tomography angiography (CTA) revealed a proximal descending thoracic aortic diameter of 37 mm and a distal infrarenal aortic diameter of 22 mm. The minimum right EIA diameter measured 6.8 mm, with an iliac tortuosity index (*X*) of 1.48 (Fig. 1C). No significant calcifications of the iliac axis were observed. The distance between the celiac trunk (CT) and the superior mesenteric artery (SMA) was 19 mm, the distance between the CT and the right renal artery (RRA) was 34.3 mm, whereas the distance between the CT and the left renal artery (LRA) was 39 mm (Fig. 1A, B). These anatomical features were compatible with the off-the-shelf *E*-nside TAAA multi-branch stent-graft system (Artivion Inc., Kennesaw, GA, USA). Its configuration allows the treatment of type I TAAA without the need of a bifurcated stent-graft, thereby preserving distal infrarenal abdominal aortic coverage and maintaining the patency of lumbar arteries, reducing the risk of spinal cord ischemia (SCI). The Cook ZDEG 42–34-160 mm and *E*-nside 38–30-222 mm were selected for treatment, over the Zenith t-Branch Thoracoabdominal Endovascular Graft by Cook Medical Inc. (Bloomington, Ind, USA) or custom-made device. Although the narrow EIA represented an exclusion criterion for advancing the grafts, TEVAR and iB-EVAR were planned without a surgical conduit, which was reserved as a bailout procedure in case of difficulty advancing the sheaths or stent-grafts. A surgical cutdown of the right CFA (primary site) and left axillary artery were performed, as a percutaneous access to the left CFA. Once adequate visualization of the right CFA was achieved, the vessel was punctured, and a 6 F introducer sheath was inserted. Systemic full heparinization was performed according to body weight, with an activated clotting time of 250 to 300 s. Multiple serial dilations with Gore Dryseal sheaths (W.L. Gore & Associates, Flagstaff, AZ, USA) 18, 20, and 22 F were carefully performed due to the small caliber of the artery. The thoracic stent-graft was deployed first, ensuring proximal stability and sealing below the left subclavian artery. The EIA accommodated both the insertion and the retrieval of the 22 F sheath (8.5 mm required), as well as the insertion and the proper deployment of the *E*-nside stent-graft (24 F sheath, 8.2 mm required). SMA and RRA were successfully incorporated with balloon-expandable Gore Viabahn VBX (W.L. Gore & Associates, Flagstaff, AZ, USA) as bridging stents, whereas catheterization of the CT was unsuccessful due to severe stenosis. The cumulative time of target vessels' catheterization was up to one hour and half. Due to the extension of the aortic coverage (≥28 cm), temporary aneurysm sac perfusion (TASP) [1] was considered to achieve spinal vessel conditioning and minimize the risks of SCI, as well as the withdrawal of the delivery system. Under fluoroscopic guidance, the sheath was pulled back to the common iliac artery by using the contralateral access to assess the integrity of the vessel. With some difficulty, the sheath was pulled farther down the EIA. At this point, we encountered some unexpected resistance, and the patient became hemodynamically unstable. A contralateral angiogram showed the right EIA rupture. The left sheath was upgraded to a long 12 F, and a 32 mm Coda balloon (Cook Medical Inc., Bloomington, Ind, USA) was placed in the distal portion of the aortic stent-graft to control blood flow to the right iliac artery (Fig. 2A). The patient's hemodynamics stabilized thereafter. Because pulling the sheath back was difficult, traumatic arterial injury was suspected and immediately confirmed by the visualization of the artery attached to the delivery system of the stent-graft (Fig. 2B). An urgent surgical conversion was performed via median laparotomy with prosthetic iliac-to-femoral bypass. The final angiogram revealed excellent flow and no extravasation of dye (Fig. 3A). The patient was transferred to the Intensive Care Unit (ICU). No complications occurred during the post-operative period and the patient was discharged from the hospital 7 days later. After one month, the patient underwent a control CTA, which showed loss of patency of the CT due to its severe stenosis and loss of patency of the LRA due to the improper apposition of the stent-graft's fabric at the ostium. Both inner branches of the LRA and CT remained patent, leading to perfusion of the aneurysmal sac. To achieve complete aneurysm exclusion, the branch cuffs were subsequently occluded using vascular plugs [2]. No signs of permanent or transient SCI were noted during the perioperative period and follow-up. After three years, control CTA confirmed the shrinkage of the aneurysmal sac, with no evidence of endoleaks (Fig. 3C). Informed written consent of patient was obtained. Patient gave her consent to the publication.

## Discussion

The risk of iatrogenic injuries to the iliac arteries during complex endovascular procedures (e.g, B-EVAR) has significantly decreased over time due to increased surgeon experience, procedural refinements, and comprehensive preprocedural planning with CTA. Although largely under-reported [3], [4], these complications (ranging from minor intimal tears to severe ruptures) remain clinically significant due to their potential to cause life-threatening hemorrhage and limb ischemia. Despite their relatively low incidence (4.3 to 15.9%) [5], [6], [7], [8], [9], [10], they seem to occur with near-equal frequency in the femoral and iliac arteries, with rare involvement of the distal aorta. While dissections account for most femoral artery injuries, ruptures predominantly affect the iliac arteries. Their occurrence is facilitated by well-known anatomical challenges and is associated with greater blood loss [10], longer operative time, extended ICU- and hospital stay, higher costs, and increased 1-year mortality [11].

Thus, in cases of challenging iliac-to-femoral anatomies, adjunctive procedures (e.g., iliac conduit) [12] appear essential for optimizing outcomes. Ideally, an iliac conduit should be planned preoperatively rather than as an emergent response to arterial disruption. However, a recent meta-analysis demonstrated that periprocedural mortality was significantly higher in patients undergoing iliac conduit [13], [14] correlating with an increased incidence of systemic complications of up to 32% [15], including bleeding, cardio-vascular and pulmonary events. Therefore, the decision for an iliac conduit must be carefully considered to ensure safe devices insertion while mitigating the risks of local and systemic complications.

Moreover, patients with TAAA often present with multiple comorbidities, severe frailty [16], and extensive atherosclerotic disease frequently affecting the access routes, making a fully endovascular approach both appropriate and challenging [17]. In such cases, adjunctive maneuvers may help mitigate the risks of complications associated with the large-bore sheath required for of the off-the-shelf devices; however, no standardized techniques are currently available.

The existing literature on iliac artery injuries is limited and primarily focuses on cases occurring after transcatheter aortic valve replacement (TAVR) [18]. Notably, Koren O et al. [19] demonstrated that significant predictors of vascular complications following TAVR include the number of curves in the iliofemoral axis and its angulation, calcifications involving more than 50% of the lumen, a sheath-to-femoral artery ratio (SFAR) of ≥1.05, and a Cedars-Sinai Index (CSI) score > 100. Additionally, Pozolo CG et al. [20] recently published a systematic review analyzing 39 full-text studies, including those on vascular interventions. The authors critically assessed the data to establish a consensus definition for large-bore access, challenging anatomy, and adjunctive or alternative approaches to the standard transfemoral technique. Their findings suggest that adjunctive maneuvers (e.g., intravascular lithotripsy, endoconduit) can reduce morbidity, mortality, procedural duration, and overall healthcare costs. Nonetheless, despite the importance of careful risk assessment, standardized and widely adopted surgical protocols remain unavailable, particularly for vascular procedures.

In our case, multiple risk factors and unfavorable anatomical characteristics were present (e.g, female gender, narrow iliac axes, and SFAR >1.20). However, the absence of significant calcifications, combined with the patient's severe comorbidities, led us to forgo the use of an iliac conduit as the primary treatment in favor of endovascular/hybrid adjuncts. Nonetheless, iliac-to-femoral arteries avulsion occurred. We believe this complication resulted from a combination of concurrent factors. Difficulties encountered during catheterization of the CT prolonged operative time and extended the duration of the delivery system's presence within the artery, likely contributing to tenacious adhesion between the endothelium and the sheath. Additionally, thoracic stent-graft insertion might have induced damage to the EIA wall, which was further exacerbated by subsequent insertion and manipulation of the *E*-nside stent-graft.

The removal of the delivery system may also have played a prominent role; retraction of the tip into the sheath may have engaged the vessel wall at its narrowest point, leading to the complete avulsion around the device. The artery was curled at the interface between the tapered tip and the sheath (Fig. 2B), suggesting mechanical strain induced by the spatial discontinuity at the gap, potentially exacerbated by prolonged operative time. To investigate this adverse event further, the delivery system was sent to the manufacturer to confirm its operational status.

The decision to forgo an iliac conduit also raises important considerations. While intended to reduce peri-operative complications, the resulting avulsion underscores the needing for individualized treatment plans and thorough preoperative risk assessment. This case highlights the potential for complications arising from unforeseen interactions between delivery systems and the vessel wall, despite technical adjuncts aimed at optimizing outcomes.

Suitable iliac/femoral access requires specific anatomical characteristics, but real-world data indicate that women are significantly less likely than men to meet instruction-for-use (IFU) criteria [21], [22], [23]. Only 34.1% of women have at least one EIA large enough to accommodate a 24 F sheath, compared to 90.1% of men [24]. Therefore, a thorough risk assessment, including frailty status [16], gender [19], [25], and hostile anatomical features, is essential to identify patients who may benefit from alternative surgical, endovascular or hybrid strategies aimed at reducing systemic and access-site-related complications.

Alternative strategies may include a surgical cutdown to CFA to facilitate retroperitoneal mobilization of the EIA and straightening of the redundant iliac artery. Additionally, multiple serial dilations using sheaths, with a 24 F introducer sheath in place (capable of accommodating the device), combined with continuous retrograde flushing with saline solution, as well as early sheath removal, are critical for minimizing device-arterial wall interactions, reducing the risk of endothelial damage.

Furthermore, improvements in delivery systems and sheaths, including more tapered tips, enhanced hydrophilic coatings, reduced device diameter, and improved trackability, are preferable to overcoming aorto-iliac limitations and reducing the reliance on iliac conduit and their associated complications.

Systematic reviews and meta-analyses focusing on vascular interventions involving large-bore devices are essential for accurately determining the rate of access-related complications during complex EVAR procedures. Additionally, they could play a crucial role in validating the risk assessment process and standardized adjunctive measures identified in TAVR studies, including their application in vascular surgery.

## Conclusion

A challenging iliac-to-femoral axis remains one of the most common pitfalls in complex endovascular aortic aneurysm repair. Preoperative imaging analysis is essential not only to anticipate difficult access in patients with narrow arteries but also for predicting prolonged operative times and potential interactions between the graft and arterial walls. Well-documented risk factors substantially increase the likelihood of EIA injuries, underscoring the importance of balancing patient health status, adverse anatomical characteristics, and treatment options. Alternative technical strategies such as surgical cutdown to CFA, retroperitoneal mobilization of the EIA with serial dilations, and continuous retrograde flushing with saline solution, could serve as standardized surgical protocols to systematically assess and address these anatomical challenges. Additionally, modifications to delivery systems may further optimize procedural outcomes. The author also assessed that the risk of ilio-femoral adherence and avulsion can be minimized with smaller sheaths, periodic sheath rotation, and early sheath removal.

## CRediT authorship contribution statement

**Antonio Marzano:** Conceptualization, Validation, Writing – review & editing, Investigation. **Enrico Sbarigia:** Funding acquisition, Resources, Supervision, Validation, Writing – original draft. **Carola D'Amico:** Investigation, Visualization. **Vincenzo Brizzi:** Resources, Visualization. **Ombretta Martinelli:** Investigation, Writing – original draft, Data curation. **Simone Cuozzo:** Conceptualization, Data curation, Investigation, Methodology, Project administration, Software, Validation, Writing – original draft.

## Consent

Informed written consent of patient was obtained. Patient gave her consent to the publication.

## Funding

No funding was provided.

## Declaration of competing interest

The authors declare that they have no competing interests.
